# Impact of the rural pipeline in medical education: practice locations of recently graduated family physicians in Ontario

**DOI:** 10.1186/s12960-017-0191-6

**Published:** 2017-02-20

**Authors:** Elizabeth F. Wenghofer, John C. Hogenbirk, Patrick E. Timony

**Affiliations:** 1 School of Rural and Northern Health, Laurentian University, 935 Ramsey Lake Rd., Sudbury, Ontario P3E 2C6 Canada; 2 Centre for Rural and Northern Health Research, Laurentian University, Sudbury, Ontario Canada

**Keywords:** Medically underserved areas, Rural practice, Family practice, Canada, Social responsibility, Northern Ontario School of Medicine

## Abstract

**Background:**

The “rural pipeline” suggests that students educated in rural, or other underserviced areas, are more likely to establish practices in such locations. It is upon this concept that the Northern Ontario School of Medicine (NOSM) was founded. Our analysis answers the following question: Are physicians who were educated at NOSM more likely to practice in rural and northern Ontario compared with physicians who were educated at other Canadian medical schools?

**Methods:**

We used data from the College of Physicians and Surgeons of Ontario. We compared practice locations of certified Ontario family physicians who had graduated from NOSM vs. other Canadian medical schools in 2009 or later. We categorized the physicians according to where they completed their undergraduate (UG) and postgraduate (PG) training, either at NOSM or elsewhere. We used logistic regression models to determine if the location of UG and PG training was associated with rural or northern Ontario practice location.

**Results:**

Of the 535 physicians examined, 67 had completed UG and/or PG medical education at NOSM. Over two thirds of physicians with any NOSM education were practicing in northern areas and 25.4% were practicing in rural areas of Ontario compared with those having no NOSM education, with 4.3 and 10.3% in northern and rural areas, respectively. Physicians who graduated from NOSM-UG were more likely to have practices located in rural Ontario (OR = 2.57; *p* = 0.014) whereas NOSM-PG physicians were more likely to have practices in northern Ontario (OR = 57.88; *p* < 0.001).

**Conclusions:**

NOSM education was associated with an increased likelihood of practicing in rural (NOSM-UG) and northern (NOSM-PG) Ontario.

## Background

The recruitment and retention of physicians to rural and remote communities is a worldwide challenge [1]. In Ontario, these challenges have led to maldistribution and a relative shortage of physicians in rural communities, particularly in the north [2]. Northern Ontario has a land mass covering over 800 000 km^2^ representing 88% of the province but with just 6% of the population (775,000 residents) [3]. While only 34% of the population is classified by Statistics Canada as rural, the remainder of the population dwell in eight urban centers, separated by 150 to 450 km of wilderness. Many of the urban areas and almost all of the rest of northern Ontario are medically underserved [4].

Rural communities can pose challenges to health care providers. For example, poorer health status of residents, fewer health care providers, lower diversity of provider specialties, and greater distance from advanced health care services mean that providers often need to assume multiple roles and complex workloads while dealing with a lack of financial resources and professional isolation [5, 6]. Many attempts have been made to attract physicians to rural and northern communities including offering financial incentives, “marketing” the rural and northern lifestyles to physicians, and enhancing medical practice support to reduce the demands placed on these providers [7]. However, these attempts have been largely unsuccessful, resulting in high turnover rates of physicians who return to the urban south once the financial incentives have run out or when the northern lifestyle has lost its appeal [8].

Educating and training medical students and residents in rural areas has been suggested as an alternative recruitment method [7, 9–11]. A compelling body of research, including research in Ontario, has demonstrated that rural training can lead to future rural practice, [12–18] particularly for individuals who have grown up in a similar location [9, 16, 19]. As Farmer and colleagues [20] explain, individuals with extensive rural experience are more likely to adapt to the culture of a rural community. This phenomenon, termed the “rural pipeline” [21], was the philosophy that underpinned the creation of the Northern Ontario School of Medicine (NOSM) [22]. A main goal of NOSM’s socially accountable mission is to increase the number of physicians in rural and northern Ontario by recruiting students from, and training them in, rural and northern regions [23, 24]. NOSM is the newest medical school in North America in over 30 years and accepted its first class of undergraduate medical students in 2005 [22]. NOSM is the joint initiative of Laurentian University and Lakehead University with two campuses in Sudbury and Thunder Bay located 1000 km apart and with students in over 90 health centers and hospitals in communities across Ontario (the majority in northern Ontario) [25]. As NOSM-trained family physicians (as NOSM undergraduate students (UGs) and/or NOSM postgraduate residents (PGs)) become independent practitioners, we can explore the contribution of the NOSM northern and rural educational experience to the rural pipeline effect on practice location. In this study, we address the following question: Are NOSM UGs and NOSM PGs more likely to locate their practices in rural and/or northern Ontario than physicians who received their training from other Canadian medical schools?

## Methods

### Setting and population

We compared NOSM UGs and PGs with the *full population* of College of Family Physicians of Canada (CFPC)-certified physicians who completed UG education at a Canadian medical school since 2009 (coinciding with NOSM’s first graduating class), had an active independent practice registration status with the College of Physicians and Surgeons of Ontario (CPSO), and had a primary practice address in Ontario in 2013. International medical graduates were excluded because of the greater potential diversity of their UG medical education.

### Study design

We used a retrospective cross-sectional design to investigate the practice location of the first two cohorts of NOSM graduates. All data were anonymized and then extracted from the CPSO 2013 Annual Membership Renewal survey and CPSO register. The physicians’ primary practice location was used to categorize location into rural/urban and north/south (described below).

UG medical school, PG medical school, and gender were extracted from the CPSO register. Year of graduation from medical school and year of birth were also extracted and used to calculate age at graduation from UG medical school. Date of birth and gender are the only demographic data collected by the CPSO.

Four *medical school categories* were created to describe a physician’s educational experience:

1. *NOSM/NOSM*: physicians who graduated from NOSM UG medical education and NOSM PG family medicine residency training. Note that physicians who completed any NOSM PG studies were considered as a NOSM PG.
2. *NOSM/Other*: those physicians who graduated from the NOSM UG program and obtained PG training elsewhere in Canada.
3. *Other/NOSM*: those who graduated from a Canadian UG program other than at NOSM and completed NOSM PG training. Note that physicians who completed any NOSM PG studies were counted as a NOSM PG.
4. *Other/Other*: physicians who did not graduate from the NOSM UG program nor complete any NOSM PG training. Physicians in this category received all of their UG and PG education from other medical schools in Canada.

The primary practice address six-character postal code was linked to Canadian census geographic areas (census subdivisions) using Statistics Canada Postal Code Conversion Files [26]. Practices located in census metropolitan areas (CMAs) and census agglomerations (CAs) were considered urban. CMAs and CAs have populations of at least 100 000 and 10 000, respectively. All areas outside of CMAs and CAs were classified as rural. We categorized a physician’s practice as northern if it fell within the jurisdiction of the North East (NE) or North West (NW) Local Health Integration Networks (LHINs) [27].

### Outcomes and analysis

The main outcome was the primary practice location of physicians in each UG/PG medical school category. We tested for differences among medical school category for age at graduation (ANOVA with least significant difference (LSD) post hoc analyses of means), gender, and the rural/urban north/south practice location classification (Fisher’s exact tests). If the test was significant (*p* ≤ 0.05), then we examined the adjusted standardized Pearson residuals to determine which cell had an observed count higher or lower than the expected count, with the expected count estimated from column and row totals.

Backwards elimination logistic regression models were used to determine if the independent variables, NOSM UG (yes vs. no) and NOSM PG (yes vs. no), were associated with the dependent variables: (i) rural/urban Ontario practice location (rural = 1; urban = 0) or (ii) north/south Ontario practice location (north = 1; south = 0). To account for the potential differences in having all, some, or none of the UG or PG education at NOSM, an interaction term between UG and PG was also entered into the model. In addition, gender and age at graduation were included in the model as covariates as both have been shown in the literature to be significantly associated with practice location [2].

Ethical approval for this study was provided by the Laurentian University Research Ethics Board (certificate number 2014-05-06), and data were extracted through an agreement with the CPSO.

### Availability of data and materials

Conditions of our ethical approval and our data sharing agreement with the College of Physicians and Surgeons of Ontario preclude sharing of data.

## Results

The population consisted of 535 family physicians (FPs), which included 67 (12.5%) who had some NOSM education, either at both UG and PG (NOSM/NOSM), at UG only (NOSM/Other), or at PG only (Other/NOSM) (Table 1). Mean age at graduation of the population was 31.9 (SD = 3.8) years. FPs in the two categories with NOSM UG were significantly older than FPs in the two Other UG categories (ANOVA *p* < 0.001 and LSD post hoc tests). Overall, 60.0% (321) of FPs were female and there were no significant differences among medical school categories (Fisher’s exact test *p* = 0.91).

**Table 1:** Study population characteristics (*n* = 535)

Medical school category	Mean age in years (SD)	Percentage of females per category (*n*)	Total percentage (*n*)
NOSM/NOSM	35.8 (6.7)^a^	55.6 (20)	6.7 (36)
NOSM/Other	36.6 (7.6)^a^	64.3 (9)	2.6 (14)
Other/NOSM	32.2 (4.0)^b^	64.7 (11)	3.2 (17)
Other/Other	31.5 (3.0)^b^	60.0 (281)	87.5 (468)
Total	31.9 (3.8)	60.0 (321)	100 (535)
Statistical test	ANOVA *p* < 0.001	Fisher’s exact test *p* = 0.91	

^a,b^Means with different superscript letters were significantly different from one another based on post hoc LSD tests

Most FPs practiced in urban (87.9%, 470/535) or southern (87.9%, 470/535) Ontario (Table 2). Proportions were lowest in the rural north (3.7%, 20/535) and highest in the urban south (79.4%, 425/535). Over two thirds (67.2%, 45/67) of graduates with any NOSM education were located in northern Ontario compared with 4.3% (20/468) of those who graduated elsewhere. Additionally, 25.4% (17/67) of those with any NOSM education were in rural Ontario compared with 10.3% (48/468) of those who graduated elsewhere.

**Table 2:** Study population by 2013 Ontario primary practice location (*n* = 535)

Location	Percentage of physicians in each location by medical school category (*n*)*				Total by location (*n*)
	NOSM/NOSM	NOSM/Other	Other/NOSM	Other/Other	
Rural north	25.0 (9)⬆	0	0	2.4 (11)⬇	3.7 (20)
Rural south	8.3 (3)	28.6 (4)⬆	5.9 (1)	7.9 (37)	8.4 (45)
Urban north	63.9 (23)⬆	7.1 (1)	70.6 (12)⬆	1.9 (9)⬇	8.4 (45)
Urban south	2.8 (1)⬇	64.3 (9)	23.5 (4)⬇	87.8 (411)⬆	79.4 (425)
Total by medical school	100 (36)	100 (14)	100 (17)	100 (468)	100 (535)

Arrow indicates that the observed count was significantly higher (⬆) or lower (⬇) than the expected count based on adjusted residuals

*Fisher’s exact test = 206.225; *p* < 0.001

Fisher’s exact test revealed significant differences in FP practice locations by medical school category (*p* < 0.001). The NOSM/NOSM group had the largest proportion of FPs practicing in rural northern Ontario of any medical school category (25%), a close second-largest proportion in urban northern Ontario (63.9%), and only 2.8% in urban southern Ontario. The NOSM/Other group had significantly more FPs located in the rural south (28.6%). The NOSM/Other group also had a large proportion of physicians practicing in the urban south (64.3%), though this was not significantly different from the overall proportion. Significantly more FPs in the Other/NOSM category were practicing in urban northern Ontario (70.6%) and fewer in urban southern Ontario. The Other/Other group was overwhelmingly located in the urban south (87.8%) and proportionally lower in northern Ontario (rural or urban).

FPs who graduated from the NOSM UG medical program were significantly more likely to have practices located in rural settings (OR = 2.6, 95% CI = 1.2–5.4, *p* = 0.014) (Table 3). FPs who were older upon graduation from their UG medical education program were significantly more likely to have practices in rural Ontario (OR = 1.11, 95% CI = 1.04–1.018, *p* = 0.001).

**Table 3:** Significant logistic regression models for family physicians’ practice location by undergraduate or postgraduate medical school

Variables*	OR	95% CI	*p* value	Nagelkerke *R* ^2^	Percentage of cases correctly classified: model with constant, final model
Rural (vs. urban) practice location in Ontario				0.093	87.9, 87.9
NOSM UG	2.57	1.21–5.44	0.014		
Age at graduation	1.11	1.04–1.18	0.001		
Northern (vs. southern) Ontario practice location				0.547	87.9, 94.4
NOSM PG	57.88	18.21–183.98	<0.001		
UG × PG	3.20	0.73–14.11	0.124		
Gender	1.89	0.87–4.09	0.106		

*Backwards elimination (conditional method) logistic regression started with gender, age at graduation, UG school (NOSM or not), PG school (NOSM or not), and the UG × PG interaction with probability for elimination set at 0.15 (*n* = 535). Final models are significant at *p* < 0.001

FPs who completed any NOSM PG training were significantly more likely to have practices in northern Ontario (OR = 57.7, 95% CI = 18.2–184.0, *p* < 0.001) (Table 3). Backwards elimination did not identify any other significant predictors of rural practice. The interaction between UG and PG medical school was not significant in the rural/urban or north/south models, though the interaction remained in the north/south model at *p* = 0.124. Gender also remained in the north/south model at *p* = 0.106.

## Discussion

This preliminary study of the first two cohorts of NOSM graduates finds support for the rural pipeline phenomenon, which suggests that students who are educated in rural and other underserviced areas are more likely to select work settings in such areas. Although we are unable to state that there is a causal association, this study does present evidence that graduating from NOSM’s UG medical education program is associated with a higher likelihood of practicing in rural Ontario. This might be partly attributed to NOSM’s undergraduate admission criterion that preferentially selects medical students with rural Canadian backgrounds [28]. The literature suggests that a distributed community-engaged medical education model, like NOSM’s, is likely to maintain or perhaps even increase the odds that medical students will practice in rural locations [14] given that it ensures that students and residents are learning within the northern Ontario context [28].

In addition, there is evidence that participation in NOSM’s postgraduate residency training program is associated with a higher likelihood of FPs practicing in northern Ontario. The literature supplies ample evidence of the positive association of PG residency training location and subsequent practice location. For instance, data on the northern Ontario family medicine residency programs that predated NOSM show that 57% of FPs (including international medical graduates) practiced in northern Ontario 2 years after completing residency in 1993–2002 [13]. Across Canada, 37–97% of FPs who had completed PG training in 2001 in a Canadian medical school were still practicing in the same province after 2 years [29]. The combination of UG and PG medical education at NOSM proportionately showed the strongest association with a rural northern Ontario practice location and was a close second to the positive association demonstrated by Other/NOSM for a practice location in urban northern Ontario. Similarly, studies from Memorial University of Newfoundland [16, 30] have shown that, relative to completing only an undergraduate medical education (at Memorial University), participation in both undergraduate medical education and postgraduate residency training was associated with a higher likelihood of practicing in the province or in rural areas. An expanded study to examine all rural-focused medical programs across Canada would provide a broader base of evidence for promoting rural practice.

### Limitations

Our results are only based on the first two cohorts of students who completed their UG or PG medical education at NOSM and were certified as FPs in Ontario. Cell size is small particularly for FPs in the NOSM/Other and Other/NOSM categories. The charter class was older, had more life experience, and had prior health care experience and many were already established in the north (e.g., the physician and/or his/her spouse/partner was previously or currently employed in northern Ontario), so some of the differences among medical school categories may disappear or strengthen as more cohorts are added. An ongoing tracking study is examining the association between other independent variables such as additional demographic characteristics (e.g., rural or northern background, marital status, social economic status) and other factors believed to influence practice characteristics (e.g., location, scope of practice), as well as recruitment and retention. A qualitative component will examine specifics of the medical education experience to investigate reasons for the choice of practice location of NOSM graduates and how the intended UG and PG program impacts may be improved. Furthermore, the tracking study will extend the current study to include subsequent cohorts and other medical specialties such as pediatrics and internal medicine. A complementary study [31] expanded the geographic coverage by examining practice location to all provinces of Canada for three cohorts of NOSM-educated family physicians. Another limitation of the study is that the time frame does not tell us much about retention, and subsequent studies will be needed to see if FPs are staying in rural or northern Ontario. Additionally, though the data used in this analysis is collected by the CPSO as part of their certification and yields a nearly perfect response rate of physicians, it is not without its limitations. The CPSO limits the type of data collected to that which is necessary for certification; thus, certain demographic information, which might help explain the results seen here, is missing from the data. Furthermore, conditions of our ethical approval and our data sharing agreement with the CPSO prevent us from linking their data with other datasets.

## Conclusions

This study provides evidence that the first two cohorts of NOSM-educated FPs were more likely to have primary practices located in northern and rural Ontario, thus offering support for the theory of the rural pipeline for medical education. As more and more NOSM graduates enter independent practice, it will be essential to monitor if these practice location trends continue. In addition, it is important to investigate the effectiveness of the rural pipeline in other health care providers (e.g., dieticians, physician assistants) who are educated under a similar conceptual framework in other NOSM-administered programs [32, 33]. Finally, and perhaps most importantly, there is a need to evaluate over time if both NOSM medical and other NOSM health provider graduates have high rates of retention in these rural and northern regions.

## Abbreviations

CA: Census agglomeration
CFPC: College of Family Physicians of Canada
CMA: Census metropolitan area
CPSO: College of Physicians and Surgeons of Ontario
FP: Family physician
LHIN: Local Health Integration Network
NOSM: Northern Ontario School of Medicine
PG: Postgraduate
UG: Undergraduate
